# Discrimination based on gender identity and decision-making regarding HIV/STI-protected sex, a cross-sectional study among trans and non-binary people in Germany

**DOI:** 10.1186/s12889-024-20464-2

**Published:** 2024-10-31

**Authors:** Mario Martín-Sánchez, Kathleen Pöge, Alexander Hahne, Jonas Hamm, Viviane Bremer, Uwe Koppe, The TASG-study group, Max Appenroth, Mine Pleasure Bouvar Wenzel, Né Fink, Silvia Rentzsch, Manuel Ricardo Garcia, Christoph Schuler, Chris Spurgat, Heinz-Jürgen Voß

**Affiliations:** 1 Department of Infectious Disease Epidemiology, Robert Koch Institute (RKI), Berlin, Germany; 2 ECDC Fellowship Programme, Field Epidemiology path (EPIET), European Centre for Disease Prevention and Control (ECDC), Stockholm, Sweden; 3 Department of Epidemiology and Health Monitoring, Robert Koch Institute (RKI), Berlin, Germany; 4 Freelance Sexual Counsellor and Bodyworker, Hamburg, Germany; 5 Deutsche Aidshilfe, Berlin, Germany; 6 Robert Koch Institute, Seestr. 10, 13353 Berlin, Germany

**Keywords:** Gender identity, Sexual health, Social discrimination, Sexual empowerment, Sexually transmitted Diseases/Infections

## Abstract

**Background:**

Trans and non-binary people are often discriminated against. Discrimination has a negative impact on health and may affect sexual health and behavior. We explored the relationship between discrimination based on gender identity and the perceived ability to make decisions about their sex life to feel as protected as desired from HIV and sexually transmitted infections (STI) among trans and non-binary people in Germany. Secondarily, we assessed whether feeling unable of making HIV/STI-protected sex decisions was associated with behaviors related to increased HIV/STI risk.

**Methods:**

We conducted a cross-sectional study using data from the Sexual Health and HIV/STI in Trans and Non-Binary Communities (TASG) survey conducted online between March-July 2022 among trans and/or non-binary people aged 18 years and older living in Germany. We described the prevalence of frequent discrimination based on gender identity. We calculated prevalence ratios (PR) with 95% confidence intervals (95% CI) for the associations between frequent experienced discrimination based on gender identity and feeling unable of making HIV/STI-protected sex decisions, and between feeling unable of making HIV/STI-protected sex decisions and behaviors related to increased HIV/STI risk.

**Results:**

Among 3077 participants, 22% reported frequent discrimination based on gender identity. Participants experiencing such discrimination reported 1.4 times more often to feel unable to make HIV/STI-protected sex decisions (PR 1.4, 95% CI 1.1–1.8). This perceived inability was associated with increased prevalence of sex under drug influence (PR 2.9, 95% CI 2.3–3.7) and condomless penetrative sex with multiple partners without PrEP (PR 2.0, 95% CI 1.4–2.9).

**Conclusion:**

Feeling unable to make decisions to feel protected from HIV/STI among trans and non-binary people was associated with both frequent discrimination and behaviors that increase the HIV/STI risk. Strategies for empowering trans and non-binary people to assert their sexual decision-making needs should be explored.

**Supplementary Information:**

The online version contains supplementary material available at 10.1186/s12889-024-20464-2.

## Background

Since 2006, legislation in Germany prohibits discrimination based on gender identity in certain areas of public life [1]. Nevertheless, results of the EU LGBTI II Survey, conducted in 2019 in all European Union (EU) Member States and the North Macedonia and Serbia, show that 66% of trans people in Germany have personally felt discriminated against due to being trans in at least one area of life in the previous 12 months [2]. When it comes to using health or social services, 40% reported experiences of discrimination, which was higher than in most EU countries [2].

Perceived discrimination has been linked to negative mental and physical health outcomes and it may also impact sexual health [3, 4]. In line with the minority stress theory, one or multiple discrimination experiences can lead to psychological distress [5], which can motivate sexual behaviors associated with higher HIV risk and other sexually transmitted infections (STI) [6–8]. Drug use has also been reported among trans and non-binary people as one way of coping with distress, which can also increase the risk to acquire HIV/STI [9]. Experienced and anticipated discrimination among trans and non-binary people has been associated with reduced access or avoidance of health services, which can limit access to HIV/STI treatment and prevention, including PrEP provision [10–12]. In addition, sexual harassment, coercion or victimization of trans and non-binary people is common [13–16]. Experiencing sexual violence has a negative impact on intimate relationships and some sexual violence survivors report an increase in sexual risk-taking [14, 17].

Literature on the sexual health of trans and non-binary people is scarce, especially for studies including non-binary people [10, 18]. In this study we used data from the “Sexual Health and HIV/STI in Trans and Non-Binary Communities” (TASG) online survey to explore the relationship between frequent discrimination based on gender identity and the perceived ability to make decisions to feel as protected as desired from HIV and STI (HIV/STI-protected sex decision-making) among trans and non-binary people in Germany. We hypothesized that experiences of discrimination might affect sexual health behaviors by reducing the perceived ability to make decisions about their sexual life. Those feeling less able to make decisions to feel protected from HIV/STI might also engage in behaviors that increase the HIV/STI risk. Therefore, as a secondary objective, we assessed whether feeling unable of making HIV/STI-protected sex decisions was associated with sexual health behaviors related to increased HIV/STI risk (past 12-month condomless penetrative sex with multiple partner without PrEP use and past 12-month sex under drug influence) and prevention (PrEP use and past 5-year use of HIV/STI counselling and testing services).

## Materials and methods

### Study design and participants

The TASG study was conceived, developed and rolled-out in a participatory way together with trans and/or non-binary representatives [19]. We conducted a cross-sectional study using an anonymous online survey between 1 March and 1 July 2022 among self-identified trans and/or non-binary people who were at least 18 years old and lived in Germany.

Before accessing the survey: (1) participants were informed of the aims of the study, the conditions of participation and the contact details of the study team, and (2) informed consent was obtained from all individual participants included in the study. Ethical approval was granted by the Berlin Medical Association [Ärztekammer Berlin] (application number: Eth-71/21).

### Procedures

The TASG online survey questionnaire included standardized survey instruments and questions developed together with community representatives (Appendix 1). It included questions on socio-demographic characteristics, gender identity, transition, sexuality, sexual behavior, HIV/STI testing and prevention, HIV status and control, mental health and experiences of discrimination. It was translated into English, French, Spanish, Russian, Farsi, Arabic and Turkish with support from community members to ensure appropriate language [20]. Participants were recruited through the project’s community representatives, community organizations, and professional associations for HIV/STI and mental health. Information about the survey was spread through QR code cards, email lists, social media, website banners, and word of mouth. To encourage participation and reduce attrition bias, participants could participate in a raffle for gift vouchers after survey completion.

During the survey promotions on social media, it attracted trans-hostile responses and calls for fake-participation. Thus, extensive data cleaning had to be conducted. The following responses were therefore excluded from the analysis: participants who made negative and hurtful comments about trans and non-binary people in free-text questions, people who did not provide any information beyond the mandatory questions (gender identity, age, size of place of residence), and responses that showed contradictory and further questionable response patterns. The plausibility of response patterns was discussed case-by-case with community members (Appendix 2).

### Measures

Frequent discrimination based on gender identity was assessed based on the question: “*How often do you experience discrimination because of your gender identity*?” Possible answers were “*always*”, “*usually*”, “*sometimes*,” and “*never*”. Responses were dichotomized and the answers “*usually*” and “*always*” were coded as frequent discrimination.

HIV/STI-protected sex decision-making was measured through a question asking for agreement to the statement: “*I can organize my sex life in such a way that I feel as protected as I want to be from HIV and STIs (e.g. by using condoms*,* PrEP).*” Responses were dichotomized in feeling able (answered “*somewhat agree*” or “*completely agree*”) and feeling unable to make HIV/STI-protected sex decisions (answered “*completely disagree*”, “*somewhat disagree*” or “*neither agree nor disagree*”).

Participant characteristics included gender identity, age group, size of place of residence, monthly available income, education level, relationship status, gender identity recognition, living according to gender identity, fulfilment of desired medical transition needs and living with HIV. Gender identity was asked using the question “*On which of the following spectrums would you most likely locate yourself?”*, possible answers were female spectrum, male spectrum, non-binary female spectrum (people placing themselves both in the non-binary and female spectrum), non-binary male (people placing themselves both in the non-binary and male spectrum) and non-binary spectrum (including abinary, polygender, genderfluid, gender nonconforming, both male and female, genderqueer, etc.). The education level was categorized using the latest school-leaving or vocational training qualification and ranged from high (e.g. master craftsman, technician, bachelor’s degree) to medium (e.g. apprenticeship, high school diploma) and low (no qualifications or up to secondary school diploma). Gender identity recognition was defined as the extent to which a person is perceived and treated according to their gender identity by others in everyday interactions. For the variable “fulfilment of desired medical transition needs”, we grouped participants in either no medical transition desired (no medical transition intervention was undertaken or desired), medical transition needs fulfilled (all desired medical transition steps had been undertaken, this could include from one to multiple transition interventions such as hormone therapy, breast, facial, vocal or genital surgery, etc.), partially fulfilled (not all desired medical transition steps had been undertaken), not fulfilled (none of the desired medical transition steps had been undertaken) and unsure (uncertain as to whether any medical transition steps were desired).

For the secondary objective, four behaviors were considered: (1) Past 12-month sex while under drug influence, assessed by the dichotomous question: “*Within the last 12 months*,* have you had sex while under the influence of drugs? (e.g. ecstasy/MDMA*,* cocaine*,* amphetamines (speed)*,* methamphetamine (crystal/meth/tina/pervitin)*,* mephedrone or ketamine).*” (2) Past 12-month condomless penetrative sex with multiple partners without using PrEP, for which responses were categorized as yes (did not always use condoms during penetrative sex, had penetrative sex with more than one person in the last 12 months and did not report PrEP use) and no (did not have penetrative sex or had it with only one person in the last 12 months or always using condoms or were PrEP users) (3). PrEP use, assessed by question “*Are you currently taking*,* or have you ever taken*,* PrEP?*”. Prep users were respondents who were currently taking PrEP or who took it on demand (4). Past 5-year use of HIV/STI counselling and testing services, assessed by the dichotomous question: “*Within the last 5 years*,* have you used an HIV/STI counselling and testing service?*”. For behaviors 2 to 4, people living with HIV were excluded from the analyses as PrEP use is not applicable, and their use of HIV/STI counseling and testing services may differ in terms of frequency or format compared to participants who were either not living with HIV or did not know of their HIV status.

### Data analysis

We described participants’ characteristics, experiences of discrimination and HIV/STI-protected sex decision-making. For categorical variables, we reported the number of respondents per category and calculated the percentage, including and excluding missing values in the denominator (valid percentage).

We compared the prevalence of frequent discrimination based on gender identity by participant characteristics using chi-squared or Fisher test. We calculated prevalence ratios (PR) with 95% confidence intervals (95% CI) for the association between frequent discrimination based on gender identity and feeling unable to make HIV/STI-protected sex decision. We compared the prevalence of sexual health behaviors related to HIV/STI exposure or prevention between those that felt able and unable to make HIV/STI-protected sex decisions, and calculated the PR and corresponding 95% CI for the association of feeling unable with each sexual behavior.

We assessed differences in participant characteristics between those with and without missing values for the variables frequent discrimination based on gender identity and HIV/STI-protected sex decision-making using chi-squared or Fisher test.

We used an alpha of 0.05 as the threshold for statistical significance in all analyses. All analyses were performed using Stata 17 software (StataCorp.2021. Stata statistical software: Release 17. College Station, TX: StataCorp LLC).

## Results

### Participant characteristics

Overall, 10,032 participants entered data in the survey. After data cleaning (Appendix 2), we included 3077 trans and non-binary people in the analysis. Most participants were aged 18–29 years (61.1%, 1880/3077) lived in a city with more than 100,000 inhabitants (61.9%, 1854/2997) and had a monthly available income inferior to 2000€ (74.5%, 1438/1930) (Table 1). Regarding relationship status, among 2626 participants, 41.1% were single, 35.5% had one steady partner and 23.4% had a different status.

**Table 1:** Participant characteristics, HIV/STI-protected sex decision-making and frequent discrimination based on gender identity among participants of the TASG study, Germany 2022

	Total (*N* = 3,077)		
	n	Percent %	Valid percent %^a^
Gender identity			
Female spectrum	677	22.0	22.0
Male spectrum	672	21.8	21.8
Non-binary female spectrum^b^	383	12.4	12.4
Non-binary male spectrum^c^	390	12.7	12.7
Non-binary	832	27.0	27.0
Other	123	4.0	4.0
Age group			
18–29 years	1880	61.1	61.1
30–39 years	771	25.1	25.1
40–49 years	271	8.8	8.8
50–59 years	126	4.1	4.1
60 years or older	29	0.9	0.9
Size of place of residence			
City with more than 100,000 inhabitants	1854	60.3	61.9
Town/City with less than 100,000 inhabitants	843	27.4	28.1
Countryside or village	300	9.7	10.0
No answer/don’t know	80	2.6	-
Monthly income			
No income	122	4.0	6.3
≤2000€	1438	46.7	74.5
>2000€	370	12.0	19.2
No answer/don’t know	1147	37.3	-
Education level			
Low	297	9.7	15.0
Medium	938	30.5	47.3
High	750	24.4	37.8
No answer	1092	35.5	-
Relationship status			
Single	1080	35.1	41.1
Steady partner	932	30.3	35.5
Other status	614	20.0	23.4
No answer	451	14.7	-
Gender identity recognition			
Yes, always	361	35.1	13.4
Sometimes/often	1584	30.3	58.8
Never	750	20.0	27.8
No answer/don’t know	382	14.7	-
Living in accordance to gender identity in daily life			
Yes	1567	50.9	53.8
Partly	1185	38.5	40.7
No	162	5.3	5.6
No answer/don’t know	163	5.3	-
Fulfilment of medical transition needs			
No medical transition desired	76	2.5	3.0
Medical transition needs fulfilled	569	18.5	22.5
Medical transition needs partially fulfilled	803	26.1	31.8
Medical transition needs not fulfilled	693	22.5	27.4
Unsure	386	12.5	15.3
No answer/not possible	550	17.9	-
Living with HIV			
Yes	17	0.6	0.7
No	2318	75.3	99.3
No answer/don’t know	742	24.1	
HIV/STI protected sex decision-making			
Feeling able	1874	60.9	87.6
Feeling unable	265	8.6	12.4
No answer	938	30.5	-
Frequent discrimination based on gender identity			
Yes	505	16.4	22.1
No	1782	57.9	77.9
No answer/don’t know	790	25.7	-
Past 12-month sex under drug influence			
Yes	247	8.0	10.1
No	2189	71.1	89.9
Missing	641	20.8	-
Past 12-month condomless penetrative sex with multiple partners without PrEP use^d^			
Yes	168	5.5	8.2
No	1870	61.1	91.8
Missing	1022	33.4	-
Current PrEP use^d^			
Yes	35	1.1	1.6
No	2173	70.6	98.4
Missing/not applicable	852	28.2	-
Past 5-year use of HIV/STI counselling and testing services^d^			
Yes	629	20.5	26.3
No	1760	57.5	73.7
Missing	671	21.9	-

^a^Percentages calculated above the total without missing values for each variable

^b^Non-binary female includes participants who located themselves both on the non-binary and on the female spectrum

^c^Non-binary male includes participants who located themselves both on the non-binary and on the male spectrum

^d^17 participants who were living with HIV were not included in these variables (*N* = 3060)

When asked to locate themselves on gender identities spectrums, 22.0% of the 3077 participants located themselves in the female spectrum, 21.8% in the male spectrum, 12.4% in the non-binary female spectrum, 12.7% in the non-binary male spectrum, 27.0% in the non-binary spectrum and 4% used other terms, but found themselves within the trans and/or non-binary spectrum. Living according to their gender identity in daily life was reported by 53.8% (1567/2914). Most participants reported that their gender identity was sometimes or often clearly recognized (58.8%, 1584/2695), while 27.8% (750/2695) answered that it was never recognized. 22.5% (569/2527) had undergone all their desired medical procedures in order to be aligned with their gender identity. A total of 17 participants (0.7%, 17/2335) who knew their HIV status were living with HIV (Table 1).

### Discrimination based on gender identity

Out of 2287 participants without missing data, discrimination based on gender identity was never experienced by 15.5%, while 62.4% experienced it sometimes, 18.8% usually and 3.3% always. Frequent discrimination (always and usually) was reported by 22.1%. Frequent discrimination based on gender identity was more prevalent among non-binary participants, participants reporting lower or no income, participants whose gender identity was never or sometimes/often recognized and participants whose medical transition needs were not met, who did not want to undergo medical transition, or who were unsure (Table 2).

**Table 2:** Participant characteristics and HIV/STI-protected sex decision-making by frequent discrimination based on gender identity among participants of the TASG study, Germany 2022

	Frequent discrimination based on gender identity		
	No (*N* = 1,782)	Yes (*N* = 505)	*p*-value
	n (%)^a^	n (%)^a^	
Gender identity			< 0.001^b^
Female spectrum	418 (84.1)	79 (15.9)	
Male spectrum	486 (88.8)	61 (11.2)	
Non-binary female spectrum	183 (70.7)	76 (29.3)	
Non-binary male spectrum	245 (78.0)	69 (22.0)	
Non-binary	407 (68.2)	190 (31.8)	
Other	43 (58.9)	30 (41.1)	
Age group			0.45^c^
18–29 years	1096 (78.5)	301 (21.5)	
30–39 years	435 (75.3)	143 (24.7)	
40–49 years	160 (80.4)	39 (19.6)	
50–59 years	79 (79.8)	20 (20.2)	
60 years or older	12 (85.7)	2 (14.3)	
Size of place of residence			0.80^b^
City with more than 100,000 inhabitants	1080 (77.8)	308 (22.2)	
Town/City with less than 100,000 inhabitants	491 (78.9)	131 (21.1)	
Countryside or village	179 (79.2)	47 (20.8)	
Monthly income			< 0.001^b^
No income	71 (71.7)	28 (28.3)	
≤2000€	924 (77.3)	272 (22.7)	
>2000€	269 (87.1)	40 (12.9)	
Education level			0.057^b^
Low	179 (73.1)	66 (26.9)	
Medium	618 (78.5)	169 (21.5)	
High	491 (80.5)	119 (19.5)	
Relationship status			0.034^b^
Single	685 (80.5)	166 (19.5)	
Steady partner	610 (78.4)	168 (21.6)	
Other status	370 (74.4)	127 (25.6)	
Gender identity recognition			< 0.001 ^c^
Yes, always	308 (94.5)	18 (5.5)	
Sometimes/often	1057 (79.1)	280 (20.9)	
Never	358 (65.0)	193 (35.0)	
Living in accordance to gender identity in daily life			< 0.001^b^
Yes	1113 (84.0)	212 (16.0)	
Partly	562 (69.4)	248 (30.6)	
No	64 (71.1)	26 (28.9)	
Fulfilment of medical transition needs			< 0.001^c^
No medical transition desired	32 (64.0)	18 (36.0)	
Medical transition needs fulfilled	433 (85.1)	76 (14.9)	
Medical transition needs partially fulfilled	573 (83.4)	114 (16.6)	
Medical transition needs not fulfilled	382 (71.1)	155 (28.9)	
Unsure	192 (74.1)	67 (25.9)	
Living with HIV			0.519^c^
Yes	10 (83.3)	2 (16.7)	
No	1495 (78.9)	401 (21.1)	
HIV/STI-protected sex decision-making			0.015^b^
Feeling able	1230 (79.0)	326 (21.0)	
Feeling unable	164 (71.9)	64 (28.1)	

^a^Percentages per row calculated above the total without missing values for each variable

^b^Chi-square test

^c^Fisher test

Participants with missing values for gender identity discrimination (25.7%, 790/3077) compared to those without had their gender identity more often not recognized, less often lived according to their gender identity and their medical transitions needs where less often fulfilled (Appendix 3).

### Discrimination based on gender identity and HIV/STI-protected sex decision-making

Feeling unable to make decisions about HIV/STI-protected sex was reported by 12.4% (265/2139). Among those feeling unable to make decisions about HIV/STI-protected sex, 28.1% (64/228) reported experiencing frequent discrimination, while 21.0% (326/1556) of those feeling able reported frequent discrimination (Table 2). This corresponds to 40% higher prevalence of frequent discrimination among those feeling unable compared to those feeling able to make HIV/STI-protected sex decisions (PR 1.4, 95% CI 1.1–1.8).

Participants with missing values in HIV/STI-protected sex decision-making (30.5%, 938/3077), compared to those without, identified themselves more often in the female spectrum, reported more frequently a low education level, where more often single, not living according to their gender identity and had their medical transitions needs less often fulfilled (Appendix 4).

### HIV/STI-protected sex decision-making and sexual health behaviors related to increased HIV/STI risk and HIV/STI prevention

Participants who felt unable to make HIV/STI-protected sex decisions reported more often past 12-month sex under drug influence (PR 2.9, 95% CI 2.3–3.7) compared with those who felt able to. Among participants not living with HIV or with unknown HIV status, feeling unable to make HIV/STI-protected sex decisions was also associated with higher prevalence of past 12-month condomless penetrative sex with multiple partners without using PrEP (PR 2.0, 95% CI 1.4–2.9), but not with PrEP use (PR 0.8, 95% CI 0.2–2.5) or past 5-year uptake of HIV/STI counselling and testing services (PR 1.0, 95% CI 0.8–1.2) (Table 3).

**Table 3:** Association between feeling unable to make decisions about HIV/STI-protected sex and sexual health behaviors related to increased HIV/STI risk and HIV/STI prevention among participants of the TASG study, Germany 2022

	Past 12-month sex under drug influence^b^			Past 12-month condomless penetrative sex with multiple partners without PrEP use^c^			PrEP use^d^			Past 5-year use of HIV/STI counselling and testing services^e^		
	No	Yes		No	Yes		No	Yes		No	Yes	
	n (%)^a^	n (%)^a^	PR (95% CI)	n (%)^a^	n (%)^a^	PR (95% CI)	n (%)^a^	n (%)^a^	PR (95% CI)	n (%)^a^	n (%)^a^	PR (95% CI)
HIV/STI-protected sex decision-making												
Feeling able	1634 (90.6)	169 (9.4)	Ref.	1410 (91.4)	133 (8.6)	Ref.	1633 (98.1)	32 (1.9)	Ref.	1245 (70.2)	528 (29.8)	Ref.
Feeling unable	176 (72.4)	67 (27.6)	2.9 (2.3–3.7)	149 (82.8)	31 (17.2)	2.0 (1.4–2.9)	201 (98.5)	3 (1.5)	0.8 (0.2–2.5)	164 (70.4)	69 (29.6)	1.0 (0.8–1.2)

*PR* Prevalence ratio, *95% CI *95% confidence intervals, *Ref* Reference category

^a^Percentages per row calculated above the total without missing values for HIV/STI-protected sex decision-making and each outcome variable

^b^Excluding 1031 participants with missing values in either this variable or HIV/STI-protected sex decision-making;

^c^Excluding 1337 participants with missing values in either this variable or HIV/STI-protected sex decision-making and 17 participants living with HIV;

^d^1191 participants with missing values in either this variable or HIV/STI-protected sex decision-making and 17 participants living with HIV;

^e^Excluding 1054 participants with missing values in either this variable or HIV/STI-protected sex decision-making and 17 participants living with HIV

## Discussion

We conducted an exploratory cross-sectional study including 3077 trans and/or non-binary people who participated in an online survey between March and July 2022 in Germany. Participants were mostly aged 18–29 and lived in cities with more than 100,000 inhabitants. We found that about one fifth of participants frequently experienced discrimination based on their gender identity. This prevalence was higher among non-binary participants. Experiencing frequent discrimination was more common among those feeling unable to make HIV/STI-protected sex decisions. Those who felt unable to make decisions about HIV/STI-protected sex more often reported sex under drug influence and penetrative condomless sex with multiple partners without using PrEP. We found no association between perceived inability to make HIV/STI-protected sex decisions and PrEP use or uptake of HIV/STI counselling and testing services.

A high prevalence of discrimination towards trans and non-binary people in Germany was also shown in previous studies: the EU LGBTI II Survey conducted in 2019 showed that 66% of the participants in Germany felt personally discriminated against in at least one of the eight different life areas during the last 12 months because of being trans [2]. The survey conducted by LesMigras between 2010 and 2011 in Germany found that the percentage of trans people experiencing discrimination was always higher than 30% across 5 different areas of life, with the workplace being the highest at 50% [21]. In our study, we observed differences in experienced discrimination across the gender identity spectrum, with a higher prevalence among non-binary participants. This differs from findings in the EU LGBTI II Survey and the 2015 U.S. Transgender Survey, where similar or lower levels of discrimination were reported by non-binary participants compared to binary trans men and women [22, 23]. However, other studies suggested that non-binary people may experience higher rates of discriminatory events compared to binary trans people [3, 24]. Non-binary individuals might disrupt cisnormative binary paradigms through their gender expressions and identities, which might subject them to higher societal stigmatization [25, 26].

Our study findings also show that discrimination against trans and non-binary people coincides with an impaired ability to engage in HIV/STI-protected sexual behaviors in line with their preferences. Several qualitative studies describe situations in which unjust or prejudicial treatment towards trans and non-binary people have had an impact in their sexual decision-making [7, 19, 27, 28]. For example, in a qualitative study among transgender people aged 13 to 24 years in the United States, participants experienced challenges with self-efficacy in sexual decision-making, particularly in interactions with male cisgender partners who often pushed for penetrative intercourse [27]. This dynamic could affect conversations about condom use or preferred sexual practices, with some participants reluctant to assert their needs [27]. Also, in the qualitative component of the TASG study in Germany, participants reported experiences of discrimination in the form of exoticization or fetishization by cisgender sexual partners [19]. Some participants perceived these experiences as a form of depersonalization, resulting in a lack of concern from their sexual partners for their protection or emotional wellbeing, while for fewer participants it was perceived as an empowering factor [19]. TASG participants also reported concerns about upsetting their sex partners, and the perceived presence of power differentials in relationships where they felt constrained in advocating for their own needs and preferences [19]. It is also important to consider that interpersonal experiences of discrimination are embedded in and linked to wider socio-structural discrimination [29]. Specifically, for trans and non-binary people societal norms and beliefs favor cisgender identities, affecting interactions for trans and non-binary people with potential dating partners, restricting spaces for socialization and contact with sexual partners, and creating harmful stereotypes about their sex lives [30, 31].

The results of our secondary objectives showed the relationship between HIV/STI-protected sex decision-making and sexual behaviors associated with higher HIV/STI risk or prevention. It is important to note that the question on HIV/STI-protected sex decision making was phrased in terms of the individual’s desired level of protection, which may vary between individuals. Despite this variation, there was a significant association between not being able to make such decisions and a higher prevalence of condomless penetrative sex with multiple sex partners without PrEP use and sex under drug influence, but not with PrEP use and use of counselling and testing services. PrEP use is important in this context, since it is a prevention strategy less dependent on partner negotiation during sexual encounters [36]. The TASG study report highlights that although 62% of individuals were aware of PrEP as a means of HIV protection, the level of PrEP use was low [19].

Our study has important strengths. First, we were able to recruit over 3000 participants from a population from which there is a paucity of sexual health information and research in Germany [10]. Second, a large number of non-binary people, who are underrepresented in the global scientific literature, participated in the study [18, 32]. Third, the participatory approach of the study that actively involved community members in the design and implementation of the study. This approach enhances the ability to accurately describe their lived realities and facilitates the translation of research findings into programs that address community needs [33].

There are several limitations to this study. First, there is a potential for selection bias due to the non-probabilistic sampling method, meaning that our participants may not represent the full diversity of trans and non-binary people in Germany. For example, those over the age of 50 may have been underrepresented, given that only a 5% of the participants belonged to that group. Second, the presence of missing values for key variables such as discrimination based on gender identity (25.7%) or HIV/STI-protected sex decision-making (30.5%). The analysis of missing data suggests that individuals with missing values on experienced discrimination based on gender identity and sexual decision-making may face higher levels of discrimination. This could imply that our results might underestimate the prevalence of discrimination based on gender identity. Third, the cross-sectional study design and question formulation did not allow to establish a causal relationship between discrimination and sexual health decision-making and we could not perform multivariate regression analysis. Prospective studies are needed to better understand the complex interplay of gender identity, transition, discrimination and sexual health over time. The TASG study did not collect information on ethnic origin/ racializing ascriptions or sexual orientation, which was decided during the participatory process of the questionnaire design. This limited our capacity to investigate the intersections of different discrimination forms.

## Conclusion

The results of the study underline that discrimination based on gender identity is commonly experienced by trans and non-binary people in Germany and differs within the gender identity spectrum. Exploring ways to enhance sexual decision-making skills among these communities may be relevant, given that feeling unable to make sexual decisions to feel as protected as desired from HIV/STI was connected to both discrimination and sexual practices associated with an increased risk of HIV/STI (sex under drug influence and penetrative condomless sex with multiple partners without using PrEP). Sexual health providers and counselling services should be aware of the potential association between discrimination and sexual health and be able to provide the necessary resources and guidance to enable informed choices about HIV/STI protection.

## Supplementary Information

Appendix 1. TASG study questionnaire.

Appendix 2. Description of the data cleaning process of the TASG study.

Appendix 3. Table A1. Comparison of participant characteristics and HIV/STI-protected sex decision-making among participants of the TASGstudy with and without missing values for discrimination based on gender identity, Germany 2022.

Appendix 4. Table A2. Comparison of participant characteristics and discrimination based on gender identity among participants of the TASGstudy with and without missing values for HIV/STI-protected sex decision-making, Germany 2022.

## Data Availability

The datasets used and/or analysed during the current study are available from the corresponding author on reasonable request.

## Abbreviations

95% CI: 95% confidence intervals
EU: European Union
PR: Prevalence ratio
STI: sexually transmitted infections
TASG study: Study "Sexual Health and HIV/STI in Trans and Non-Binary Communities"
