# Supplementary material for: Discrimination based on gender identity and decision-making regarding HIV/STI-protected sex, a cross-sectional study among trans and non-binary people in Germany
Source: BMC Public Health. 2024 Oct 31;24:3013. doi: 10.1186/s12889-024-20464-2 (PMC11526635; doi:10.1186/s12889-024-20464-2)
Supplement: Supplementary file 1 — Appendix 1. TASG study questionnaire. [file 12889_2024_20464_MOESM1_ESM.pdf]

Discrimination based on gender identity and decision-making regarding HIV/STI-protected sex, a cross-sectional study among trans and non-binary people in Germany

Appendix 1. TASG study questionnaire

1.	**TASG study: Sexual health and HIV/STIs in transgender* and non-binary communities**	
2.	Language selection	
3.	**Please select a language**	German<br>English<br>French<br>Spanish<br>Turkish<br>Arabic<br>Russian<br>Farsi
4.	**Welcome to the online survey for people from transgender* and non-binary communities**	
5.	**Survey on sexual health and HIV/STIs in transgender* and non-binary communities**<br><br>Information regarding the study<br><br>**Why are we doing this survey?**<br>In Germany, there is currently a lack of data on the sexual health of people from transgender* and non-binary communities. However, this information is an important component in being able to extrapolate requirements in the context of prevention and medical treatment. For the first time in Germany, this study aims to provide answers to the following questions:<br>1. How common are HIV/STIs in transgender* and non-binary communities in Germany?<br>2. What factors play a role in sexual risk?<br>3. What needs exist for HIV/STI prevention, counselling and medical treatment?<br><br>**What do we want to achieve?**<br>We want to identify factors and measures that will contribute to improving HIV/STI prevention and medical treatment for transgender* and non-binary people.	

	**Who can take part in the survey?**<br>To take part in the survey, you must be on the transgender* or non-binary spectrum, be over 18 years of age and live in Germany.<br><br>Participants will be asked questions about, for example, their life situation, sex life and experiences of discrimination. We understand that these are sensitive topics and will give you advance warning before asking the questions. If you feel uncomfortable with some of the questions, you can skip them. Some participants might find it helpful to have someone they trust available while answering the questions who can provide them with support if necessary.	
6.	If you have already started the survey and want to continue, please enter your PIN here:	
7.	Welcome back to the survey.<br><br>How do you want to proceed?	Continue the survey<br>Restart the survey
8.	Go to survey	
9.	**About this study**<br>This online survey is part of the “Sexual Health and HIV/STIs in Transgender* and Non-Binary Communities” (TASG) study, which is being carried out in collaboration with representatives and activists from transgender* and non-binary communities, Deutsche Aidshilfe (DAH) and the Robert Koch Institute (RKI). The funding has been provided by the German Federal Ministry of Health (Bundesministerium für Gesundheit, BMG). The study was designed in collaboration with representatives and activists from transgender* and non-binary communities, who will be involved from beginning to end.<br>**We understand that people use different terms to denote their identity. In this survey, we have agreed to use the terms transgender* and non-binary as examples. At certain points during the survey we also use example terms, e.g. for certain parts of the body. We understand that not all participants feel that these terms are correct for them or would use them for themselves. However, we have agreed on these example terms, as we cannot specify the full range of possible terms in each question.**<br>Further information on the study can be found here.<br>**Prize draw:** As a thank-you for taking part in the survey, all participants are eligible to enter our prize draw to win one of 20 vouchers	Yes<br>No

	worth €50 from xxx. Participation in the prize draw is of course voluntary. You can also take part in the study without taking part in the prize draw. Data for the prize draw is stored separately from the survey data, meaning that information from the survey cannot be linked to your contact details. Further information on the prize draw can be found at the end of the survey.<br>**Anonymous and voluntary:** Participation in the survey is anonymous unless you take part in our prize draw. If you choose to participate in the prize draw, you must provide an email address to enable us to send you a voucher in the event that you win. In this case, your participation may no longer be anonymous. However, your email address will be stored separately from your survey data, meaning that it is impossible to link your email address with your answers to the survey. In any case, your participation is voluntary. Your IP address will be saved while the questionnaire is opened. If you close the questionnaire, your IP will be deleted. By participating, you are consenting to the entries you make in this survey being used for the purposes described**.** Please make sure that any answers you enter in the free text fields <u>do not contain</u> any personal data about yourself or others**.** You can choose not to continue with the survey at any time or leave individual questions unanswered. It goes without saying that you will suffer no disadvantages if you choose not participate in this survey or decide not to continue.<br>**Duration**: The survey should take around 30 to 60 minutes to complete. You can pause and continue the survey at any time. To do this, please make a note or take a screenshot of your PIN (displayed).<br>**Contact:** xxx<br><br>**Consent to participation in the survey**<br><br>I have understood the conditions of participation in the survey and agree to participate in the study:	
10.	Questionnaire<br><br>We will start by asking you 5 questions about yourself.<br><br>We understand that there are a wide variety of terms used on the subject of sex and gender, and that people use these terms in different	____________________

	ways. In this survey, we use the term “gender identity” to describe a person's gender self-image, gender self-designation and/or sex.<br>We would like to ask you about your identity. This question all comes down your own personal feelings at this time.<br><br>What term do you use to describe your (gender) identity?	
11.	In order to enable statistical evaluation, we have to group a range of different terms into a handful of categories. In the following, we have listed some categories in which various terms are combined to form spectrum. Not all terms within a spectrum need to be relevant to you, or you may reject certain terms in relation to yourself.<br><br>On which of the following spectrums would you be most likely to locate yourself?	**Female spectrum** (woman, transfeminine, transgender* woman, MtF, transsexual woman etc.<br><br>**Male spectrum** (male, transmasculine, transgender* male, FtM, transsexual male etc.)<br><br>**Non-binary, female spectrum** (both non-binary and female spectrum)<br><br>**Non-binary, male spectrum** (both non-binary and male spectrum)<br><br>**Non-binary spectrum** (abinary, polygender, genderfluid, gender non-conforming, both male and female, genderqueer etc.)<br><br>I use other terms, but I can basically assign the generic term transgender* and/or non-binary to myself<br><br>I do not locate myself on the transgender* or non-binary spectrum
3	How old are you?	Younger than 18<br>18-29<br>30-39<br>40-49<br>50-59<br>60 or older
4	Which description applies to where you currently live?	Metropolis (over 1 million inhabitants)<br>City (up to 1 million inhabitants)<br>Town (up to 100,000 inhabitants)<br>Small town (up to 20,000 inhabitants)<br>Countryside/village (up to 5,000 inhabitants)<br>I live in changing accommodation in different places<br>No answer

		Don't know
5	In which region do you live?<br><br>Please enter the first three digits of the postcode of the place where you have mainly lived during the last 6 months.<br><br>Examples:<br><br>If your postcode is 04103, please enter 041.<br><br>If your postcode is 10777, please enter 107.<br><br>If you don't want to say where you live, please enter 000.	Numerical answer<br>3 numbers "_ _ _XX"
6	The next section deals with the topic of "transition". We understand that this is a sensitive subject that people experience in a very individual way.<br>After consulting with people from the transgender* and non-binary communities, we decided to include some questions on this subject in the study. The background for this decision is that sexual health and, for example, sexually risky behaviour can differ during different phases of your life.<br><br>This section contains 9 questions on the subject. Depending on your answers, you may be asked up to 7 follow-up questions. If there is a question you don't want to answer, simply click on "Next" to skip to the next question.	
7	What age range were you in when you first realised that you could be transgender* and/or non-binary?	Between the ages of _ _ and _ _<br><br>error message:<br><br>Please enter a value/number between 0 and 99.
8	Were there phases during which you tried to live as the sex you were assigned at birth while you were aware that you were located on the transgender* or non-binary spectrum, or were supressing this awareness?	Yes<br>No<br>No answer
9	How old were you when you first told someone that you were probably transgender* or non-binary?	_ _ years old<br>So far, I haven't shared my gender identity with anyone.<br><br>error message:<br><br>Please enter a value/number between 0 and 99.

10	Do you live in accordance with your gender identity in your everyday life?	Yes<br>Partly (e.g. only on certain days, at certain times or in certain places)<br>No<br>No answer<br>Don't know
11	Have you taken steps in your everyday life to be perceived in accordance with your gender identity? If so, which?<br><br>Please select all the answers which apply.<br><br>If you have taken no steps of this type, simply click "Next".	I have shared my gender identity with other people<br>I am using a new or modified first name<br>I am using adapted pronouns<br>I have adjusted my gender expression (e.g. behaviour, voice, gait, clothing)<br>I have made non-operative physical adjustments (e.g. shaving, hairstyles, wigs/hair pieces, tattoos, packing, tucking, binding, epithesis, piercing, cosmetics)
12	Have you undergone one or more of the following medical procedures in order to adjust your gender identity, or are you planning to do so in the future?<br><br><br>Hormone therapy<br>Operations in the chest area (e.g. mastectomy, breast augmentation)<br>Genital surgery<br>Epilation (hair removal) or hair transplantation<br>Surgery on the vocal cords or larynx<br>Facial surgery<br>Silicone/implants/transplantation of body fat to change your body shape<br>Speech therapy	I have done this already<br>I plan to do this in the future<br>Not sure if I want to do this<br>I don't want this<br>Not possible (e.g. for medical, financial, religious, family or social reasons)<br>Does not apply (e.g. beard epilation for transgender* men)<br>No answer
13	Have you ever been administered or received hormones for transitioning in the form of injections?	Yes<br>No<br>No answer
14	Re-using syringes or needles can put you at risk of contracting HIV and other blood-borne diseases.<br><br>Have you injected hormones with used syringes or needles that people gave you, lent you or sold you?	No<br>Yes, sometimes<br>Yes, every time<br>No answer<br>Don't know
15	Have you completed one or both of the following legal procedures in order to adjust your gender identity, or are you planning to do so in the future?<br>• Changing your first name<br>• Changing your civil status (e.g. your legally assigned sex)	Yes, I have done this<br>I'm in the process of doing this<br>No, but I'm planning to do it in the future<br>No, I'm still thinking about it<br>No, I don't want to change anything

		No, I can't change my first name/civil status (e.g. unrecognised first names, entry as "non-binary", or ongoing asylum proceedings)
16	Have you completed your change of civil status before 2011?	Yes<br>No<br>No answer<br>Don't know
17	People can perceive their gender identity as corresponding more or less with their body. In your own personal perception: How satisfied are you with your body in terms of your gender identity?	Very satisfied<br>Somewhat satisfied<br>Neither satisfied nor dissatisfied<br>Somewhat dissatisfied<br>Very dissatisfied<br>No answer<br>Don't know
18	In everyday life it is possible that people are not recognized or treated according to their gender identity because the people in their environment cannot place or doesn't respect it. In general, how often is your gender identity clearly recognised?	Never<br>Sometimes<br>Mostly<br>Always<br>No answer<br>Don't know
19	In general, how often is your gender identity respected?	Never<br>Sometimes<br>Mostly<br>Always<br>No answer<br>Don't know
20	How often do you experience discrimination because your gender identity?	Never<br>Sometimes<br>Mostly<br>Always<br>No answer<br>Don't know
21	In the next section, we are interested in individual aspects that prevent or have prevented you from making legal and/or medical adjustments to your gender identity.<br><br>Which obstacles do you envisage or have you encountered on your journey to physical and/or legal transition?<br><br>Financial situation (e.g. costs for transition measures, expert opinions or court costs)<br><br>Employment situation (e.g. lack of time off, fear of losing your job/apprenticeship position), (fear of) workplace discrimination	No obstacles<br>Was an obstacle in the past<br>Currently an obstacle<br>Was an obstacle in the past and currently still an obstacle<br>Does not apply<br>No answer

	Access to the health system (e.g. no health insurance, no secure residential status)<br><br>Lack of supportive or medically qualified doctors, therapists, clinics etc.<br><br>Discrimination when dealing with practitioners (doctors, therapists, clinics, etc.)<br><br>Guidelines in statutory health insurance companies (Medizinische Dienste der Krankenversicherung, MDK) (e.g. diagnoses, the idea of two sexes as the norm)<br><br>Social situation (e.g. religious, cultural, familial obstacles)	
22	In the next section of the survey, we will ask you 9 questions about your relationships, sexuality and sexual health. Depending on your answers, you may be asked up to 8 follow-up questions.<br><br>We understand that questions about sexuality are very intimate and some people feel uncomfortable answering them. Nevertheless, these questions are an important part of our study, as there is currently no data available on the sexual health of people from transgender* and non-binary communities in Germany. We hope that, with your help, we can fill this gap.<br><br>If you don't want to answer a certain question, simply click on "Next" or select the "No answer" option.	
23	What is your current relationship status?<br><br>We are aware that your relationship status does not have to be synonymous with your lived sexuality. Therefore, we have included further questions about sexuality.	I'm single<br>I have a steady partner<br>I have several steady partners / I live a polyamorous lifestyle<br>I have one or more casual partnerships<br>I'm not sure<br>It's complicated<br>No answer
24	Do you have sex with other people outside of your partnership(s)?	No<br>Yes<br>No answer<br>Don't know
**25**	Do(es) your partner(s) have sex with other people outside of your partnership?	No<br>Yes<br>No answer<br>Don't know
26	Do you agree with the following statements?<br><br>I have a sex life.<br><br>I feel sexually desired	Completely disagree<br>Somewhat disagree<br>Neither agree nor disagree<br>Somewhat agree<br>Completely agree

		No answer
27	On a scale from 1 to 10 (1: completely dissatisfied; 10: completely satisfied), how satisfied are you with your sex life?<br><br>If you don't want to answer this question, simply click on "Next".	[Please select]<br>1 (completely dissatisfied)<br>2<br>3<br>4<br>5<br>6<br>7<br>8<br>9<br>10 (completely satisfied)
28	Do you agree with the following statements?<br><br>I can organise my sex life in such a way that I feel as protected as I want to be from HIV and STIs (e.g. by using condoms, PrEP).<br><br>I only have sex with partners with whom I feel as safe as I want.<br><br>I only have sex in places where I feel as safe as I want.<br><br>I feel safe during sex because I can decide for myself when and whether to come out as transgender* or non-binary.<br><br>It's easy for me to express my needs and help shape the sex I have.<br><br>It's easy for me to say "no" to sex that I don't want.<br><br>I can say "no" to sex without incurring any disadvantages.	Completely disagree<br>Somewhat disagree<br>Neither agree nor disagree<br>Somewhat agree<br>Completely agree<br>No answer
29	People can live their sexuality in different ways. In the following section, we are interested in sexual encounters which involve mutual contact of mucous membranes. This includes rubbing mucous membranes together as well as oral sex (e.g. licking, sucking) and penetration (e.g. fucking).<br><br>It doesn't matter whether barriers (e.g. condoms, dental dams) are used.<br><br>For this question, we are not counting French kissing (e.g. kissing with tongues) as sexual contact.<br><br>Within the last 12 months, have you had any sexual encounters that match the description above?	Yes<br>No<br>Don't know

30	Within the last 12 months, with how many different people have you had sexual encounters that resulted in mucous membrane contact?	0<br>1<br>2-3<br>4-5<br>6-10<br>11-20<br>More than 20<br>Don't know
31	Sex that involves penetration (e.g. fucking) can play a particular role in the transmission of certain STIs, such as HIV.<br><br>This does not include penetrative sex with external aids such as strap-ons or externally attached genitalia.<br><br>In the past 12 months, have you had sex that resulted in penetration?	Yes<br>No<br>Don't know
	Within the last 12 months, with how many different people have you had penetrative sex (e.g. fucking)?	0<br>1<br>2-3<br>4-5<br>6-10<br>11-20<br>More than 20<br>Don't know
32	Within the last 12 months, have you had sex while under the influence of drugs?<br>(e.g. ecstasy/MDMA, cocaine, amphetamines (speed), methamphetamine (crystal, meth, tina, pervitin), mephedrone or ketamine)	Yes<br>No
33	Have you ever injected drugs or allowed someone to inject you with drugs?	No<br>Yes
34	Within the last 12 months, how often have you injected or been injected with drugs?	Once<br>Twice<br>3 times<br>4 times<br>5 times<br>6 times<br>7 times<br>8 times<br>9 times<br>10 times<br>11 times<br>12 times or more
35	When was the last time you injected drugs with a used syringe or needle that someone gave you, lent you or sold you?	Never<br>Within the last 7 days<br>Within the last 4 weeks<br>Within the last 6 months<br>Within the last 12 months<br>Within the last 5 years<br>More than 5 years ago<br>No answer<br>Don't know
36	Some people make use of sex workers or engage in sex work themselves. Since this can be a part of people’s lived sexuality, we have included it in this survey.	Yes<br>No<br>No answer

	Within the last 12 months, have you paid for sex?<br><br>By payment we mean the exchange of money, gifts or favours (housing, groceries, etc.) for sex.	
37	Within the last 12 months, have you been paid for sex?<br><br>By payment we mean the exchange of money, gifts or favours (housing, groceries, etc.) for sex.	Yes<br>No<br>No answer
38	Why were you paid to have sex/why do you engage in sex work?<br><br>Please select all the answers that apply:	I want to decide my own working environment<br><br>I feel empowered through sex work<br><br>I feel desired through sex work<br><br>I have no other way of making money/earning a living<br><br>I have no other way of finding housing<br><br>It pays better than other jobs<br><br>Because I am forced to<br><br>Don't know<br>No answer
39	The next section deals with counselling and testing for HIV and other sexually transmitted infections (STIs). We would like to ask you 5 questions on this topic. Depending on your answers, you may be asked up to 4 follow-up questions.<br><br>Within the last 5 years, have you wanted advice about HIV/STIs or testing for HIV/STIs?	Yes<br>No<br>Don't know<br>No answer
40	Do you know one or more places which offer counselling and HIV/STI testing especially for transgender* and non-binary people?	Yes<br>No<br>Don't know<br>No answer
41	How long does it take you to get to the nearest HIV/STI counselling and testing facility for transgender* and non-binary people?	Up to 30 minutes<br>Up to 60 minutes<br>Up to 120 minutes<br>Up to 180 minutes<br>More than 180 minutes<br>It’s too far away for me<br>Don't know<br>No answer

42	Within the last 5 years, have you used an HIV/STI counselling and testing service?	Yes<br>No<br>No answer
43	Why haven't you used an HIV/STI counselling and testing service?<br><br>Please select all the answers that apply:	There are no services near me<br>The waiting list for an appointment is too long<br>I expected or have already experienced discrimination/stigmatisation<br>I was too scared<br>I didn't know that it's important for me<br>I haven't had sex/no risk<br>Don't know
44	What was the <u>last</u> HIV/STI counselling and testing service that you used?	Checkpoint/Aidshilfe<br>Local health authority<br>Family doctor/GP<br>Specialist medical practice/HIV practice<br>HIV self-tests<br>Send-in tests<br>Other HIV/STI counselling and testing service, namely: [free text]
45	Was the last HIV/STI counselling and testing you used explicitly aimed at people on the transgender* or non-binary spectrum?	Yes<br>No<br>Don't know<br>No answer
46	During your most recent experience of HIV/STI counselling or testing, how satisfied were you with the support and information you received from the facility in question?	Very satisfied<br>Somewhat satisfied<br>Neither satisfied nor dissatisfied<br>Somewhat dissatisfied<br>Very dissatisfied<br>Don't know<br>No answer
47	When you think of **the last place you had** HIV/STI counselling or testing, to what extent were the following criteria met?<br><br>You were given the option to enter your names, pronouns and gender identity correctly when registering (online)<br><br>You were asked for your correct names, pronouns and gender identity at the beginning of the conversation/during the initial contact (including acceptance of non-binary pronouns such as "they" or the desire not to use pronouns)<br><br>Staff used respectful attitudes and language (e.g. you were asked which terms should be used for body parts and genitals and these terms were used during your conversation).<br><br>Your gender identity and sexual or romantic orientation were not questioned; staff were open to different or new worlds	Completely applies<br>Mostly applies<br>Sometimes applies, sometimes doesn't<br>Doesn't really apply<br>Does not apply at all<br>I can't rate this

	Staff demonstrated a sex-positive attitude without judgement regarding your partners, friends, experiences from the community and/or engagement in sex work<br><br>Your individual HIV/STI counselling and testing needs were addressed<br><br>Staff had received anti-racism & awareness training and demonstrated sensitivity for those who have experienced racism as well as BIPOC representatives (black, indigenous, people of colour)<br><br>There was the option to arrange multilingual consultations/translation<br><br>The facility had information on HIV/STIs tailored to transgender* and non-binary people (e.g. taking hormones and PrEP at the same time)<br><br>Staff were accepting and interacted sensitively with people of all religions (e.g. Judaism, Islam, etc.)<br><br>Staff dealt with the different sexual risks and life situations which may apply to individual transgender* or non-binary people (e.g. stigmatisation/discrimination by (cisgender) sex partners)<br><br>Staff were accepting and handled people's sexual needs in a positive manner, regardless of age<br><br>Staff had no preconceived assumptions about genital shape, sexual behaviour or gender identity<br><br>Facility provided barrier-free access to counselling (e.g. wheelchair-accessible rooms and sanitary facilities, aids for the visually impaired, visually and acoustically low-irritation environments)<br><br>Facility provided supported communication (simplified or easy language, pictures, PCs, videos, puppets)<br><br>Staff had knowledge of the needs of people with chronic illnesses, e.g. depression<br><br>Facility offered peer counselling by people who are not only transgender* or non-binary, but also belong to other groups that are affected by discrimination	

	Facility provided gender-neutral spaces (e.g. unisex toilets, individual toilets, changing rooms)<br><br>Staff were aware of the often highly stressed/emotional relationship with medical institutions	
48	In the next 7 questions, we will ask you questions about how often you have been tested for HIV and sexually transmitted infections (STIs) and the results of your tests. Depending on your answers, you may be asked up to 2 follow-up questions.	
49	You have stated that you have made use of HIV/STI counselling or testing services within the last 5 years.<br><br>Within the last 5 years, how often have you been tested for HIV and/or other sexually transmitted infections such as syphilis?<br><br>HIV<br>Syphilis (lues)<br>Gonorrhea (tripper, the clap)<br>Chlamydia<br>Hepatitis C<br>Human papillomavirus (HPV test/pap smear)	At least once every 6 months<br>At least once a year<br>At least once every 2 years<br>At least once every 5 years<br>Never<br><br>When I need to<br>Don't know
50	What do you know about how HIV is transmitted?<br>The following two statements are APPLICABLE.<br>Did you know?<br><br>HIV cannot be passed on through kissing because saliva does not transmit HIV.<br><br>If an HIV-positive person is receiving effective treatment and their viral load is undetectable, they cannot transmit HIV through sexual encounters.	I knew that<br>I wasn’t sure about that<br>I didn't know that<br>I don’t understand that<br>I don’t believe that’s true<br>No answer
51	Have you ever been diagnosed as HIV positive?	Yes<br>No<br>Don't know<br>No answer
52	What do you know about sexually transmitted infections (STIs)? STIs include, for example, syphilis, gonorrhea (tripper) and chlamydia.<br><br>The following three statements are APPLICABLE.<br>Did you know?<br><br>Most STIs are more easily transmitted than HIV.	I knew that<br>I wasn’t sure about that<br>I didn't know that<br>I don’t understand that<br>I don’t believe that’s true<br>No answer

	STIs are often symptom-free and you can be infected without realising it.<br><br>The correct use of barriers such as condoms, gloves, foils or dental dams during<br>sex reduces the likelihood of passing on an STI (or HIV) to others.	
53	Have you ever been diagnosed with or tested positive for one or more of these sexually transmitted infections (STIs)?<br><br>Please select all the answers that apply:	Syphilis (lues)<br>Gonorrhea (tripper, the clap)<br>Chlamydia<br>Hepatitis B<br>Hepatitis C<br>Human papillomavirus (HPV test/pap smear)<br>Trichomoniasis<br>None<br>Don't know
54	When was the last time you were diagnosed with this STI?<br><br>Syphilis (lues)<br>Gonorrhea (tripper, the clap)<br>Chlamydia<br>Hepatitis C<br>Trichomoniasis	Within the last 12 months<br>More than a year ago but less than 2 years ago<br>More than 2 years ago but less than 3 years ago<br>3 years ago or longer<br>Don’t know<br>No answer
55	When was the first time you were diagnosed with the following STIs?<br><br>Hepatitis B<br>Human papillomavirus (HPV)	Within the last 12 months<br>More than a year ago but less than 2 years ago<br>More than 2 years ago but less than 3 years ago<br>3 years ago or longer<br>Don’t know<br>No answer
56	People with a short urethra are sometimes at higher risk of certain infections, such as inflammation of the urinary bladder. People with short urethras include, for example, people with a front hole, vagina, pussy, etc. Here we are referring to people who haven’t (yet) had gender reassignment surgery as well as people who have had gender reassignment surgery.<br><br>Are you someone who has a short urethra?	Yes<br>No<br>Don't know
57	Within the last 12 months, how often have you had bladder infections or urinary tract infections?	Never<br>Once<br>Twice<br>3 times<br>4 times<br>5 times<br>6 times<br>7 times<br>8 times<br>9 times<br>10 times<br>11 times<br>12 times or more

58	In the next section, we will ask you 10 questions about potential precautions and strategies against HIV and sexually transmitted infections (STIs). Depending on your answers, you may be asked up to 11 follow-up questions. The questions will refer to the use of barriers as well as PrEP (HIV pre-exposure prophylaxis), PEP (HIV post-exposure prophylaxis) and vaccinations against hepatitis A and B.	
59	What precautions against HIV and other sexually transmitted infections (STIs) are you currently using to protect yourself?<br><br>Please select all answers that apply to you	I use condoms (internal or external condoms).<br>I use dental dams.<br>I use gloves.<br>I have sex with people who have proved that they don't have STIs or HIV.<br>I use PrEP (pre-exposure prophylaxis).<br>I have sex with people who are HIV negative and who are taking PrEP.<br>I have sex with people who are HIV positive and who are below the viral load detection limit.<br>I avoid situations with a high risk of infection (e.g. sex under the influence of drugs).<br>I get tested regularly.<br>I use PEP (post-exposure prophylaxis).<br>I avoid sexual contact.<br>I avoid certain sexual practices (e.g. penetration).<br>I don't (or very rarely) change sexual partners.<br>Other:_____
60	What precautions against sexually transmitted infections (STIs) are you currently using to protect yourself?<br><br>Please select all answers that apply to you	I use condoms (internal or external condoms)<br>I use dental dams<br>I use gloves<br>I have sex with people who have proved that they don't have STIs<br>I avoid situations with a high risk of infection (e.g. sex under the influence of drugs)<br>I get tested regularly<br>I avoid sexual contact<br>I avoid certain sexual practices (e.g. penetration)<br>I don't (or very rarely) change sexual partners<br><br>Other:_____
61	How often do you use condoms during genital penetrative sex?	Always<br>Often (approx. 75% of the time)<br>About half the time (approx. 50%)

	By genital penetrative sex we mean sexual practices in which the genital mucous membranes are involved in penetration (e.g. fucking). This also includes anal intercourse. It does not include, for example, genital penetration with strap-ons/externally attached genitals or oral sex.	Sometimes (approx. 25% of the time)<br>Never<br>Don't know<br>No answer
62	How often do you use dental dams or condoms for oral sex?	Always<br>Often (approx. 75% of the time)<br>About half the time (approx. 50%)<br>Sometimes (approx. 25% of the time)<br>Never<br>Don't know
63	Have you ever heard of PrEP?	Yes<br>No<br>Not sure<br>No answer
64	Did you know?<br><br>The following three statements are APPLICABLE.<br><br>In HIV pre-exposure prophylaxis (PrEP), an HIV-negative person takes tablets before and after sex to protect themselves from HIV.<br><br>PrEP is approved for use in the form of a daily tablet.<br><br>The effects of hormones are not affected by PrEP.	I knew that<br>I wasn’t sure about that<br>I didn't know that<br>I don’t understand that<br>I don’t believe that’s true<br>No answer
65	Have you ever tried to get PrEP (regardless of whether you were ultimately prescribed it or took it)?	Yes<br>No<br>Don't know<br>No answer
66	Have you ever received PrEP?	Yes<br>No<br>Don't know<br>No answer
67	Are you currently taking, or have you ever taken, PrEP?	Yes, I’m currently taking PrEP every day<br>Yes, I take PrEP at certain times when I need it (on demand or daily during certain periods.<br>Yes, I have taken PrEP once, but I am no longer taking it<br>No<br>Don't know<br>No answer
68	Why don’t you use PrEP?<br><br>Please select all the answers that apply:	My risk of HIV infection is not high enough.<br>I am HIV positive.<br>I can’t take PrEP for medical reasons.<br>I can't find a doctor who will prescribe PrEP for me.

		I don't want to talk to my doctor about my sex life.<br>Regular visits to the doctor and regular testing is too much effort for me.<br>Taking a pill every day is too stressful for me.<br>I am afraid of negative reactions from others/sexual partners.<br>I use other safer sex methods.<br>I am afraid of side effects.<br>I am afraid of interactions with my hormone treatment.<br>I don't trust doctors.<br>I get conflicting information about taking it.<br>Other reason: _______
69	Where did you get your PrEP medication?<br><br>(please state the most recent source from which you obtained PrEP)	From a German pharmacy – my health insurance covers the costs<br>From a German pharmacy – I pay for it myself with a private prescription<br>From friends/acquaintances<br>From a dealer<br>At a sex party<br>By taking part in a clinical study<br>From the Internet/online ordering from abroad<br>I regularly travel to another country where I can get PrEP<br>I used PEP medication as PrEP<br>Other source: _________
70	Why don't you get PrEP through your health insurance?<br><br>Please select all the answers that apply:	I didn't know it was an option.<br>I can't find a doctor who will prescribe PrEP for me through health insurance.<br>My health insurance does not reimburse the costs of PrEP.<br>It's more convenient for me if I don't have to go to the doctor. (informal users only)<br>I don’t have health insurance.<br>I’m afraid of being stigmatised.
71	When you are taking or have taken PrEP, how often do you use condoms during genital penetrative sex?	Always (more than 95% of the time)<br>Often (approx. 75% of the time)<br>About half the time (approx. 50%)<br>Sometimes (approx. 25% of the time)<br>Never<br>Don't know
72	Why have you stopped taking PrEP?<br><br>Please select all the answers that apply:	I feel safe enough with other protective measures (condoms, etc).<br>I am at low risk of HIV infection right now, so I don't need PrEP.

		Taking a pill every day is too stressful for me.<br>I had side effects.<br>I'm afraid of the long-term side effects of PrEP.<br>I've had too many sexually transmitted infections while taking PrEP and not using condoms during sex.<br>I am having trouble finding a doctor who will prescribe PrEP for me.<br>I'm afraid of being stigmatised by others or by my sex partner.<br>I found out I was HIV positive.<br>To me, using PrEP is immoral and/or irresponsible.<br>I don't want to expose my body to unnecessary chemicals.<br>I don't feel sufficiently protected by PrEP.<br>I was concerned that PreP might interact badly with my hormone treatment.<br>Other reason: _______
73	Would you take PrEP again in the future?<br><br>Please select all the answers that apply:	Yes, if my risk of HIV infection increased.<br>Yes, if I can find a doctor who will prescribe PrEP for me.<br>Yes, as the costs for PrEP and the associated tests are now covered by my health insurance.<br>Yes, if I feel well informed about the risks and potential side effects (e.g. in connection with my hormone treatment).<br>No<br>Don't know<br>No answer
74	If PrEP made sense for you and you could find a doctor to prescribe it, how likely would you be to take PrEP?	Very unlikely<br>Fairly unlikely<br>I'm not sure<br>Fairly likely<br>Very likely<br>Don't know<br>No answer
75	Did you know?<br><br>The following three statements are APPLICABLE.<br><br>Post-exposure prophylaxis (PEP) tries to prevent HIV infection after exposure to the virus (e.g. after unprotected contact with the mucous membrane).<br><br>PEP involves taking an HIV treatment for one month.	I knew that<br>I wasn't sure about that<br>I didn't know that<br>I don't understand that<br>I don't believe that's true<br>No answer

	You should start taking PEP as soon as possible after exposure to risk, preferably within a few hours.	
76	Have you ever tried to get PEP (regardless of whether you were ultimately prescribed it or took it)?	No<br>Yes<br>Don't know<br>No answer
77	Have you ever been treated with PEP?	No, I have never been treated with PEP<br>No, I could have received PEP, but I decided against it<br>Yes, I have been treated with PEP exactly once<br>Yes, I have been treated with PEP more than once<br>Don't know<br>No answer
78	The following five statements are APPLICABLE.<br>Did you know?<br><br>'Hepatitis' is inflammation of the liver.<br><br>Most of the time, hepatitis is caused by viruses.<br><br>There are different types of hepatitis named after the letters of the alphabet (A, B, C, D, E, ...).<br><br>There are vaccinations for hepatitis A and hepatitis B.<br><br>There is no vaccination for hepatitis C.	I knew that<br>I wasn’t sure about that<br>I didn't know that<br>I don’t understand that<br>I don’t believe that’s true<br>No answer
79	Have you been vaccinated against hepatitis A?	No, I’m immune to hepatitis A because I’ve already had it<br>No, and I don’t know if I’m immune or not<br>Yes, I am fully vaccinated<br>Yes, but I’m not fully vaccinated<br>Don't know<br>No answer
80	Have you been vaccinated against hepatitis B?	No, I’m immune to hepatitis B because I’ve already had it<br>No, and I don’t know if I’m immune or not<br>No, I suffer from chronic hepatitis B<br>Yes, I am fully vaccinated<br>Yes, but I’m not fully vaccinated<br>Yes, but vaccination was unsuccessful<br>Don't know<br>No answer
81	Do you know where you can get vaccinated against hepatitis A and B?	Yes<br>No

		I’m not sure
82	We would like to find out more about how you protect yourself from HIV and other sexually transmitted infections and how this may have changed over time.<br>You have indicated that you do not live in accordance with your gender identity, but identify yourself as being on the transgender* or non-binary spectrum.<br><br>Compared to your current situation: Before you became aware that you identify yourself as being on the transgender* or non-binary spectrum, did you have unprotected sex more frequently (e.g. sex without a condom or PrEP with different partners)?	I had unprotected sex more frequently than I do now<br>I had unprotected sex less frequently than I do now<br>I had unprotected sex as frequently as I do now<br>I didn’t have sex then<br>Don't know<br>No answer
83	You have indicated that you live partially or permanently in accordance with your gender identity.<br><br>Compared to your current situation: Before you started to live in accordance with your gender identity, did you have unprotected sex more frequently (e.g. sex without a condom or PrEP with different partners)?	I had unprotected sex more frequently than I do now<br>I had unprotected sex less frequently than I do now<br>I had unprotected sex as frequently as I do now<br>I didn’t have sex then<br>Don't know<br>No answer
84	You have indicated that there were one or more phases in your life in which you tried to live as the sex you were assigned at birth while you were aware that you were located on the transgender* or non-binary spectrum, or were supressing this awareness.<br>Compared to your current situation: Did you have unprotected sex more frequently during this/these phase(s) (e.g. sex without a condom or PrEP with different partners)?	I had unprotected sex more frequently than I do now<br>I had unprotected sex less frequently than I do now<br>I had unprotected sex as frequently as I do now<br>I didn’t have sex then<br>Don't know<br>No answer
85	You have indicated that you have been diagnosed as HIV positive. In the following section, we would like to ask you 7 questions about your HIV diagnosis and treatment. Depending on your answers, you may be asked up to 5 follow-up questions.<br><br>When were you first diagnosed with HIV?	Drop-down menu with years from 1984-2022
86	Where did you first get a positive test result for HIV?	Family doctor/GP practice<br>Other specialist medical practice/HIV practice<br>Outpatient hospital visit<br>Inpatient hospital visit<br>Another HIV testing facility e.g. local health authority, Aidshilfe, Checkpoint)<br>Donating blood<br>Took your own blood sample and sent it away for analysis

		Did a home test and got your own result<br>Bar/pub, club or sauna<br>Mobile testing site<br>Somewhere else: [free text]
87	When was the last time you visited a doctor to check the progress of your HIV infection?<br><br>(Please let us know when you last did this, even if it was an exception for you.)	Never<br>Within the last 24 hours<br>Within the last 7 days<br>Within the last 4 weeks<br>Within the last 6 months<br>Within the last 12 months<br>Within the last 5 years<br>More than 5 years ago<br>Don't know<br>No answer
88	What was your viral load level at your last check-up?	Undetectable<br>Detectable<br>Don't know<br>No answer
89	Have you ever received anti-HIV drug treatment (ART, HAART, combination treatment)?	Yes<br>No<br>Don't know
90	How long did it take you to start HIV treatment after you were diagnosed with HIV?	_ _ years _ _ months
91	Why not?<br><br>Please select all answers that apply to you	I have only recently been diagnosed.<br>My doctor says I don't need any treatment at the moment.<br>To avoid side effects.<br>I don't think it's necessary.<br>I'm afraid other people will notice.<br>I don't want to be reminded of HIV every day.<br>I don’t have health insurance.<br>I can’t afford the cost of treatment.<br>I don't know where to get treatment.<br>I’m afraid of being stigmatised.<br>Other reason: [free text]
92	Are you <u>currently</u> receiving drug treatment for HIV?	Yes<br>No
93	Why have you stopped drug treatment?<br><br>Please select all answers that apply to you	My doctor says I don't need any treatment at the moment<br>To avoid side effects<br>I don't think it's necessary<br>I'm afraid other people will notice<br>I don't want to be reminded of HIV every day<br>I don’t have health insurance (any more)<br>I can’t afford the cost of treatment any more<br>Other reason: ____ [free text]
94	Before your HIV diagnosis,<br>did you ever try to get PrEP (regardless of whether you were ultimately prescribed it or took it)?	No<br>Yes<br>No answer
95	Before your HIV diagnosis,	No, I have never received PrEP

	did you ever receive PrEP (HIV pre-exposure prophylaxis)?	No, I could have received PrEP, but I decided against it<br>Yes, I took PrEP long term<br>Yes, I took PrEP on demand<br>Don't know<br>No answer
96	Before your HIV diagnosis,<br>did you ever try to get PEP (post-exposure prophylaxis) (regardless of whether you were ultimately prescribed it or<br>took it)?	No<br>Yes<br>No answer
97	Before your HIV diagnosis,<br>were you ever treated with PEP?	No, I have never received PEP<br>No, I could have received PEP, but I decided against it<br>Yes, I have been treated with PEP exactly once<br>Yes, I have been treated with PEP more than once<br>Don't know<br>No answer
98	In the next section, we will ask you 8 questions about potential psychological disorders, social support and inner attitudes towards your gender identity. These aspects are important for our study because they can influence sexual health.<br><br>We understand that these are sensitive issues which can trigger stressful memories and/or feelings in some participants. When answering the questions, take as much time as you need. You can pause the survey at any time and return to it later. If you don't want to answer a certain question, simply click on "Next".	
99	In general, how satisfied are you with your current life, if 0 means "not satisfied at all" and 10 means "completely satisfied"?<br><br>If you don't want to answer this question, simply click on "Next".	0 to 10
100	How often have you felt affected by the following complaints during the last 2 weeks?<br><br>Little interest or enjoyment in your activities<br><br>Depression, melancholy or hopelessness<br><br>Difficulty falling asleep, sleeping through the night, or sleeping too much<br><br>Fatigue, or the feeling of having no energy<br><br>Lack of appetite or overwhelming desire to eat	Not at all<br>On some days<br>On more than half the days<br>Nearly every day

	A poor opinion of yourself; the feeling of being a failure or a disappointment to your family<br><br>Difficulty concentrating, for example when reading the newspaper or watching television<br><br>Have your movements or speech slowed down so much that it is noticeable to others? Or, on the contrary, have you been “fidgety” or restless, and thus with a stronger urge to move than usual?<br><br>Thoughts that you would rather be dead or feelings of self-harm	Spitzer, R. L. Kroenke, K. Williams, J. B. (1999). Validation and utility of a self-report version of PRIME-MD: The PHQ primary care study. *JAMA*, *282*, 1737– 1744
101	How often have you felt affected by the following complaints during the last 2 weeks?<br><br>Nervousness, anxiety or tension<br>Not being able to stop or control your worrying<br>Worrying excessively about various matters<br>Difficulty relaxing<br>Restlessness, making it difficult to sit still<br>Feeling irritable or quick to lose your temper<br>Feeling afraid, as if something bad is about to happen	Not at all<br>On some days<br>On more than half the days<br>Nearly every day<br><br>Spitzer, R. L., Kroenke, K., Williams, Williams, J. B. W., & Löwe, B. (2006). A brief measure for assessing generalized anxiety disorder. *Archives of Internal Medicine*, *166*, 1092–1097.
102	Within the last 2 weeks, how often have you noticed or experienced the following things?<br><br>Depression<br>Exposure to racism<br>Exposure to discrimination<br>Exposure to violence<br>Exposure to marginalisation<br>Exposure to anti-Semitism<br>Loneliness<br>Panic attacks<br>Aggression<br>Uncontrollable crying/bursting into tears<br>Racing heart/palpitations<br>Isolation<br>Numbness or increased sensitivity of the body or parts of the body<br>Dissociation (feeling like you’re outside your body)<br>Increased consumption of stimulants and/or intoxicants<br>Changes in sexual behaviour (withdrawal or risky disinhibition)	Not at all<br>On some days<br>On more than half the days<br>Nearly every day
103	Do you agree with the following statements?<br><br>There are people I can count on in an emergency.<br>There are people who accept me as I am.	Completely disagree<br>Somewhat disagree<br>Sometimes agree, sometimes disagree<br>Somewhat agree

	I have people who enjoy doing the same things as me.<br>When I am ill, I have people I can ask to do important things for me without hesitation (e.g. shopping).<br>I have people who are good at listening to me when I want to express myself.<br>I have people who share my concerns and interests.<br>There are people whose help I can rely on when I need them.<br>With some people, I can be high-spirited and really let go.<br>I feel like part of a group (or groups) of people who share my views and values.	Completely agree
104	The following section is about social relationships and contacts.<br>How many people are close enough friends for you to rely on them when you have serious personal problems?	None<br>1 to 2<br>3 to 5<br>6 or more<br>Don't know<br>No answer
105	In the next question, we would like to learn more about your attitudes towards your gender identity.<br><br>Please indicate the extent to which you agree with the following statements:<br><br>My gender identity makes me feel special and unique.<br>I don't mind telling people that my gender identity is different from the sex I was assigned at birth.<br>I'm happy to talk about my gender identity with practically anyone.<br>For me it's a gift that my gender identity is different from the sex I was assigned at birth.<br>I'm like other people, but I'm also special because my gender identity is different from the sex I was assigned at birth.<br>I'm proud to be someone whose gender identity is different from the sex they were assigned at birth.<br>I have no problem revealing to other people that my gender identity is different to the sex I was assigned at birth.<br>I would prefer that people knew everything and accepted me, including my gender identity and gender history.	Completely disagree<br>Somewhat disagree<br>Neutral<br>Agree<br>Completely agree<br><br>Testa, R. J., Habarth, J., Peta, J., Balsam, K., & Bockting, W. (2015). Development of the Gender Minority Stress and Resilience Measure. *Psychology of Sexual Orientation and Gender Diversity*, *2*(1), 65–77. https://doi.org/10.1037/sgd0000081
106	Please indicate the extent to which you agree with the following statements:<br><br>My gender identity bothers me.<br>My gender identity makes me feel like a freak.	Completely disagree<br>Somewhat disagree<br>Neutral<br>Agree<br>Completely agree

	When I think about my gender identity, I feel depressed (decreased motivation, loss of interest, joylessness).<br>When I think about my gender identity, I feel unhappy.<br>Because of my gender identity, I feel like an outcast.<br>I often ask myself: Why can't my gender identity just be "normal"?<br>I find my gender identity embarrassing.<br>I envy people who don't have the same gender identity as me.	Testa, R. J., Habarth, J., Peta, J., Balsam, K., & Bockting, W. (2015). Development of the Gender Minority Stress and Resilience Measure. *Psychology of Sexual Orientation and Gender Diversity*, *2*(1), 65–77. https://doi.org/10.1037/sgd0000081
107	We would now like to ask you 5 questions regarding possible instances of discrimination or violence in your everyday life, in a medical context or in the context of potential sexual partners.<br>These questions may remind you of past experiences that were or are associated with stressful feelings. Asking these questions helps us to find out about important aspects of your living situation which can influence sexual health and risks.<br><br>When answering the questions, take as much time as you need. You can pause the survey at any time and return to it later. Before each question, we will ask you whether you want to provide information on your personal experience of the relevant topic. If you don't want to answer one or more of the questions, you can skip individual questions or the entire section.	
108	The following question deals with potential instances of discrimination or violence in everyday life.<br><br>Are you ready to answer a detailed question about your potential experience of discrimination and violence in everyday life?	Yes<br><br>No, I don't want to answer any questions about my experience of discrimination and violence in everyday life.<br><br>I want to skip this section entirely. I don't want to answer any questions about potential experiences of discrimination.
109	When was the last time you had the following experiences?<br><br>We are aware that this list is not exhaustive and does not include all aspects of exposure to discrimination and violence in everyday life. If you want, you can enter any experiences you have had which are not included here as free text at the end of the list.	Never<br>Within the last 4 weeks<br>Within the last 6 months<br>Within the last 12 months<br>Within the last 5 years<br>More than 5 years ago<br>Don't know<br>No answer

	I was belittled through looks or gestures (e.g. stared at).<br>I was insulted, called names or laughed at.<br>I was marginalised or ignored.<br>Someone threatened to leave me or break off contact with me.<br>Someone broke off contact with me.<br>Someone called me the wrong pronoun or a name that I no longer use.<br>Someone failed to take my gender identity seriously.<br>Someone told me that I am mentally ill.<br>I experienced racism (e.g. anti-black racism, anti-Muslim racism, anti-Asian racism, anti-Sinti or anti-Roma racism, anti-Slavism, racism against indigenous people, racism against migrants).<br>I experienced anti-Semitism.<br>Someone denied my German nationhood.<br>I experienced (racist) micro-aggressions (e.g. “But where are you really from?” or “You speak good German”, or my name was frequently mispronounced).<br>I was pigeonholed because of (presumed) “ethnic” affiliations.<br>I was treated insensitively.<br>I was told to seek psychotherapy to change my gender identity.<br>I was asked inappropriate questions about my body or my sexuality.<br>I was discriminated against because of my age.<br>Someone threatened to out me as transgender* or non-binary.<br>Someone outed me without asking.<br>Someone discriminated against me because of my religion/worldview or one ascribed to me.<br>I was ridiculed.<br>I was discriminated against because of my weight.<br>I was photographed or filmed against my will.<br>I was discriminated against because of my provenance/language.<br>I was threatened with violence or physically threatened.<br>I was held back by society and have suffered discrimination as a result.<br>Someone overstepped my physical boundaries/touched me.<br>I was physically attacked.<br>I received death threats.<br>Someone attributed or denied me qualities and abilities based on prejudice.<br>Other, namely: ________<br>(You can enter further experiences in the free text field)	

110	The following question deals with your experiences and the situations you've been in that regard education, work and housing, which may have possibly entailed material or financial disadvantages. These questions may remind you of past experiences that are associated with stressful feelings. When answering, take as much time as you need. You can pause the survey at any time and return to it later.<br><br>If you don't want to answer this question, you can skip this section.<br>Are you ready to answer a detailed question about your experience of being disadvantaged?	Yes<br><br>No, I don't want to answer questions about being disadvantaged.<br><br>I want to skip this section entirely. I don't want to answer any questions about potential experiences of discrimination.
111	When was the last time you had the following experiences?<br><br>We are aware that we cannot depict all aspects of being disadvantaged in this list. If you want, you can enter any experiences you have had which are not included here as free text at the end of the list.<br><br><br>I was declined for an interview or rejected for a job.<br>I was classified by the job centre as being unplaceable.<br>I was unable to complete an apprenticeship or go to school or university.<br>I was fired or told I had to leave an educational institution.<br>I received my papers/documents unchanged.<br>I received a lower salary than someone else doing a similar job.<br>I was disadvantaged because of my place of residence (e.g. refugee camp, social flashpoint).<br>I experience racism in the housing and employment market which led to me being severely disadvantaged.<br>I don't yet have the necessary language skills to be able deal with the authorities or find work.<br>I was bullied in the professional and/or educational sector or received less acknowledgement than my colleagues.<br>Offers or locations were not accessible.<br>My work performance was downgraded or rated comparatively worse than my colleagues.<br>I wasn't able to get to one or more locations.<br>I was passed over for promotions.<br>I was rejected for an apartment.	Never<br>Within the last 4 weeks<br>Within the last 6 months<br>Within the last 12 months<br>Within the last 5 years<br>More than 5 years ago<br>Don't know<br>No answer

	I was not awarded residential status.<br><br>Other, namely: ________<br>(You can enter further experiences in the free text field)	
112	The following question deals with your experiences and the situations you've been in that regard medical contexts.<br>These questions may remind you of past experiences that are associated with stressful feelings. When answering the question, take as much time as you need. You can pause the survey at any time and return to it later. If you don't want to answer questions about these experiences, you can skip this section.<br><br>Are you ready to answer a detailed question about your experiences in medical contexts?	Yes<br><br>No, I don't want to answer any questions about my experiences in medical contexts.<br><br>I want to skip this section entirely. I don't want to answer any questions about my potential experiences of discrimination.
113	When was the last time you had the following experiences in the medical sector?<br><br>I didn't take advantage of certain medical services because I was concerned that I would be treated inappropriately.<br><br>My concerns were not taken seriously.<br><br>I was under the impression that practitioners' personal views and attitude influenced diagnoses or treatment decisions.<br><br>I had to inform a practitioner about transgender or non-binary issues (e.g. medical or legal aspects, life situation).<br><br>I was asked inappropriate questions in a medical consultation or during medical treatment.<br><br>I was addressed by the wrong name or the wrong pronoun.<br><br>I was refused a diagnosis.<br><br>I received a stigmatising misdiagnosis.<br><br>I was given a diagnosis. It took me a lot of time and energy to understand that it was a misdiagnosis and to distance myself from it.<br><br>I was under the impression that hasty conclusions were drawn and premature associations were made, needs or fears were implied.	Never<br>Within the last 4 weeks<br>Within the last 6 months<br>Within the last 12 months<br>Within the last 5 years<br>More than 5 years ago<br>Don't know<br>No answer

	I was denied or offered delayed access to medical or therapeutic services, or I was not offered them at all.<br><br>I experienced racism (disadvantages when making appointments, rudeness and disrespect, health consequences caused by racism/the stress of belonging to a minority group being ignored, rough treatment, refusal of treatment due to assumed characteristics (e.g. reduced sensitivity to pain), indoctrination, refusal to issue sick notes, pain not taken seriously, misdiagnoses due to Eurocentric training (e.g. failure to recognise skin diseases in people with a different skin colour), having to justify why I had not had medical treatment in my “country of origin".).<br><br>I was told I was too fat to have an operation.<br><br>My doctors were not specialised in treating transgender and non-binary people.<br><br>I had to change doctors because I didn’t feel they were competent to deal with my issues, but they didn't want to do any training.<br><br>I was mistreated.<br><br>I was threatened that word of my case would get around among practitioners.<br><br>Other medical diagnoses were linked to my gender identity.<br><br>My identity as a transgender* or non-binary person was not taken seriously because I have a psychiatric diagnosis.<br><br>My identity as a transgender* or non-binary person was not taken seriously because I am “too young”.<br><br>My identity as a transgender* or non-binary person was not taken seriously due to racism.<br><br>My identity as a transgender* or non-binary person was not taken seriously due to my religion/worldview.<br><br>My identity as a transgender* or non-binary person was not taken seriously because I have a disability or am disabled by society.	

	I was told that I had to get a grip on other “health problems” (e.g. depression, obesity) before I could transition.<br><br>Other, namely: ________<br>(You can enter further experiences in the free text field)	
114	In the next section, we would like to find out whether you have had negative and/or discriminatory experiences in the <u>sexual context</u>.<br>These questions may remind you of past experiences that are associated with stressful feelings. When answering the question, take as much time as you need. You can pause the survey at any time and return to it later. If you don’t want to answer any more questions about your experience of discrimination, you can skip this section entirely.<br><br>Are you ready to answer a question about potential negative and/or discriminatory experiences in the sexual context?	Yes<br><br>No, I don't want to answer any questions about rejection and/or discrimination by (potential) sex partners.<br><br>I want to skip this section entirely. I don’t want to answer any questions about possible experiences of discrimination.
115	When was the last time you had one of the following experiences?<br><br>Someone refused to meet me face-to-face or cancelled a meeting.<br><br>Someone assumed that I don’t have sex.<br><br>I was made a sex object (e.g. reduced to my physical characteristics, fetishised).<br><br>Someone told me that there’s something missing from my body (e.g. body parts or fluids).<br><br>I was rejected because of my age.<br><br>I was rejected as a sex partner.<br><br>I was not recognised in my gender identity.<br><br>I was rejected because of my provenance or language.<br><br>I felt like I had to prove my gender identity through my behaviour.<br><br>I experienced racism (rejection because of my skin colour, facial features, body shape, hair, language, provenance and related stereotypes).<br><br>I was approached in a racist, fetishising way.	Never<br>Within the last 4 weeks<br>Within the last 6 months<br>Within the last 12 months<br>Within the last 5 years<br>More than 5 years ago<br>Don't know<br>No answer

	I was pressured to present myself or act in a stereotypical way.<br><br>I was rejected because of my (presumed) religion.<br><br>My sexual desires were ignored or disregarded.<br><br>I was ascribed a certain sexual behaviour (e.g. passive or active).<br><br>I was accused of lying or pretending to be someone I'm not because I did not say that I was transgender.<br><br>I was rejected because of my disability/impairment/ chronic illness.<br><br>I was insulted.<br><br>In a very short space of time, I was asked extremely intimate questions that would not be asked of a cisgender person.<br><br>I was laughed at or made fun of because of my identity or physical characteristics.<br><br>I was told that something like me doesn't or shouldn't exist.<br><br>My (potential) sexual partners unknowingly used incorrect terms.<br><br>My (potential) sexual partners used incorrect terms knowingly or out of ignorance.<br><br>Other, namely: ________<br>(You can enter further experiences in the free text field)	
116	The following question is about <u>sexual assault and sexual violence</u>. These questions may remind you of past experiences that are associated with stressful feelings.<br>When answering the question, take as much time as you need. You can pause the survey at any time and return to it later. If you don't want to answer this question, you can skip this section.<br><br>Are you ready to answer a detailed question about your potential experiences of sexual assault or sexual violence?	Yes<br><br>No, I don't want to answer any questions about my potential experiences of sexual assault or sexual violence<br><br>I want to skip this section entirely. I don't want to answer any questions about my potential experiences of discrimination.

117	When was the last time you had the following experiences?<br><br>Inappropriate comments were made about my body/parts of my body.<br><br>Due to being black/a person of colour and being transgender, I experienced violence, was exoticised, was rejected, or was not taken seriously.<br><br>I was touched against my will.<br><br>I was threatened.<br><br>I was coerced or forced into sexual acts (e.g. in return for being able to live with someone or under the threat of being abandoned).<br><br>My desire for safer sex was overridden/ignored/not acted upon.<br><br>Without prior agreement or contrary to what we had agreed, the sex was violent (e.g. I was strangled, beaten).<br><br>Sexual acts were carried out against my will.<br><br>Orifices were used during sex that I didn't want used.<br><br>Without prior agreement or contrary to what we had agreed, orifices were used during sex.<br><br>I was raped.<br><br>Other, namely: ________<br>(You can enter further experiences in the free text field)	Never<br>Within the last 4 weeks<br>Within the last 6 months<br>Within the last 12 months<br>Within the last 5 years<br>More than 5 years ago<br>Don't know<br>No answer
118	In the last section of the survey, we will ask you 8 questions about your life circumstances. Depending on your answers, you may be asked up to 2 follow-up questions. If you don't want to answer certain questions, simply click on "Next".<br><br>What is your highest school-leaving qualification?<br><br>If you didn't go to school in Germany, please give the answer that most closely corresponds to your school-leaving certificate.	I left school without a certificate<br>Volks-/Hauptschulabschluss (elementary/lower secondary school leaving certificate/GDR year 8)<br>Realschulabschluss/Mittlere Reife (intermediate/general secondary school leaving certificate/GDR year 10)<br>Fachhochschulabschluss (technical college certificate)<br>Abitur (general qualification for university entrance)<br>Other school leaving qualification: [free text]<br>I am currently at school/in vocational training

		Don't know<br>No answer
119	Which vocational training or technical/university degrees do you have?<br><br>If you didn't receive vocational training or go to technical college/university in Germany, please give the answer that most closely corresponds to your qualifications.	In-house vocational training (apprenticeship)<br>Master craftsman, technician or equivalent technical college qualification<br>Bachelor's degree<br>Master's degree, diploma, engineering degree, German state or teaching examination (e.g. studies to be a doctor, teacher, pharmacist, food chemist, psychotherapist, jurist or surveyor)<br>PhD/doctorate<br>Other professional qualification: _________<br>I am currently a trainee/apprentice<br>I am currently a student<br>I have no professional qualifications<br>Don't know<br>No answer
120	What is your current employment situation?	Working full-time<br>Working part-time (over 50%)<br>Working part-time (under 50%)<br>Working in a mini-job (€450 basis)<br>Receiving unemployment benefit (ALG I)<br>Receiving unemployment benefit (ALG II)<br>Self-employed<br>On parental leave or "maternity protection"<br>Retired<br>Not working<br>Seasonal worker<br>I don't have a work permit
121	How much money do you have at your disposal per month?<br><br>This can include money from wages, salaries, income from self-employment, pensions, unemployment benefit, housing benefit, child benefit, etc.	No income<br>From €1 to under €1000<br>From €1000 to €1500<br>From €1501 to €2000<br>From €2001 to €3000<br>From €3001 to €4000<br>From €4001 to €5000<br>Over €5000<br><br>Don't know<br>No answer
122	How do you manage on your monthly income?	Very badly<br>Badly<br>Relatively badly<br>Relatively well<br>Well<br>Very well
123	Do you have access to the German health system?	Yes<br>No<br>No answer

		Don't know
124	Why don’t you have access to the German health system?<br><br>Please select all answers that apply to you:	I don’t have health insurance.<br>I have not been awarded residential status (yet).<br>My application for asylum was rejected.<br>I can’t find accurate information on how to get access.<br>Because nearly everything is written in German and I don’t understand German very well.<br>I am homeless/I have no permanent address and therefore no health insurance.<br>Other: __________
125	Do you have children or have you taken on responsibility for children (co-parenting)?	No<br>Yes
126	Does the child/do the children live in your household?	No<br>Yes, all of the time<br>Yes, some of the time
127	Are you currently or have you ever been homeless?	Yes, I am currently homeless<br>Yes, I have been homeless in the past<br>No<br>No answer
128	Is there anything else you would like to tell us? We would be delighted to hear your comments and remarks.<br><br>Please do **<u>not</u> enter any personal data** about yourself or any other individual in this field.	
