# Supplementary material for: Discrimination based on gender identity and decision-making regarding HIV/STI-protected sex, a cross-sectional study among trans and non-binary people in Germany
Source: BMC Public Health. 2024 Oct 31;24:3013. doi: 10.1186/s12889-024-20464-2 (PMC11526635; doi:10.1186/s12889-024-20464-2)
Supplement: Supplementary file 2 — Appendix 2. Description of the data cleaning process of the TASG study. [file 12889_2024_20464_MOESM2_ESM.docx]

**Discrimination based on gender identity and decision-making regarding HIV/STI-protected sex, a cross-sectional study among trans and non-binary people in Germany**

**Appendix 2**

**Description of the data cleaning process of the TASG study**

On March 28, 2022 following a Twitter post by the RKI social media team promoting the study, there was a call to distort the study. It is feared that, especially from that date, there are participants in the dataset who may have provided incorrect information. At the same time, the community accounts urged from that date as many people as possible to participate in the study and not allow it to be compromised. Therefore, it is to be expected that, especially in the days starting on March 28, there will be a mixture of participants from the target groups and participants who provide distorted information in the study population.

The following strategies were used for data cleaning to exclude participants who do not belong to our study population. The unadjusted data set contained 10,032 records. In a first step all individuals who did not meet the participation requirements (younger than 18 years not located in the trans or non-binary spectrum) were excluded. In a second step, all participants who responded during the critical period from March 28, 2022 to April 02, 2022 were excluded if they made trans hostile or study-unrelated comments in the open-ended comment options of the questionnaire, had few data other than the mandatory items or provided identical answers in the question batteries. In a third step, further questionable and/or contradictory statements in the data were examined and all open response answers were screened for trans-hostile comments beyond the critical period. For example, one case was excluded in which the participant age was lower than 40 years old. However, it was stated that the person became aware of their gender identity at the age of 88 and that they told someone else about their gender identity at the age of 88. Not only is the age information in the case contradictory, but the number 88 is also used as a code in the right-wing extremist scene in Germany and can refer to this here. In these cases, participants were excluded from the study. In individual cases of questionable and/or contradictory response patterns but otherwise consistent data, the questionable and/or contradictory values were defined as missing but all the other information considered valid was retained. For example, questionable data included participants who reported having all sexually transmitted infections. Contradictory data concerned information on age where a participant stated that they had begun to become aware of their gender identity at the age of 30 and that this process had ended at the age of 20. In those cases, if the remaining data were consistent only these values were defined as missing.
