# Supplementary material for: Discrimination based on gender identity and decision-making regarding HIV/STI-protected sex, a cross-sectional study among trans and non-binary people in Germany
Source: BMC Public Health. 2024 Oct 31;24:3013. doi: 10.1186/s12889-024-20464-2 (PMC11526635; doi:10.1186/s12889-024-20464-2)
Supplement: Supplementary file 3 — Appendix 3. Table A1. Comparison of participant characteristics and HIV/STI-protected sex decision-making among participants of the TASGstudy with and without missing values for discrimination based on gender identity, Germany 2022. [file 12889_2024_20464_MOESM3_ESM.docx]

**Discrimination based on gender identity and decision-making regarding HIV/STI-protected sex, a cross-sectional study among trans and non-binary people in Germany**

**Appendix 3**

**Table A1.** **Comparison of participant characteristics and HIV/STI-protected sex decision-making among participants of the TASG study with and without missing values for discrimination based on gender identity, Germany 2022.**

|  | Discrimination based on gender identity | |  |
| --- | --- | --- | --- |
|  | No-missing (N=2287) | Missing  (N=790) | p-value |
|  | n (%)^a^ | n (%)^a^ |  |
| Gender identity |  |  | <0.001^b^ |
| Female spectrum | 497 (21.7%) | 180 (22.8%) |  |
| Male spectrum | 547 (23.9%) | 125 (15.8%) |  |
| Non-binary female spectrum | 259 (11.3%) | 124 (15.7%) |  |
| Non-binary male spectrum | 314 (13.7%) | 76 (9.6%) |  |
| Non-binary | 597 (26.1%) | 235 (29.7%) |  |
| Other | 73 (3.2%) | 50 (6.3%) |  |
| Age group |  |  | 0.030^c^ |
| 18-29 years | 1397 (61.1%) | 483 (61.1%) |  |
| 30-39 years | 578 (25.3%) | 193 (24.4%) |  |
| 40-49 years | 199 (8.7%) | 72 (9.1%) |  |
| 50-59 years | 99 (4.3%) | 27 (3.4%) |  |
| 60 years or older | 14 (0.6%) | 15 (1.9%) |  |
| Size of place of residence |  |  | 0.80^b^ |
| City with more than 100,000 inhabitants | 1388 (62.1%) | 466 (61.2%) |  |
| Town/City with less than 100,000 inhabitants | 622 (27.8%) | 221 (29.0%) |  |
| Countryside or village | 226 (10.1%) | 74 (9.7%) |  |
| Monthly income |  |  | 0.83^b^ |
| No income | 99 (6.2%) | 23 (7.1%) |  |
| ≤2000€ | 1196 (74.6%) | 242 (74.2%) |  |
| >2000€ | 309 (19.3%) | 61 (18.7%) |  |
| Education level |  |  | 0.38^b^ |
| Low | 245 (14.9%) | 52 (15.2%) |  |
| Medium | 787 (47.9%) | 151 (44.0%) |  |
| High | 610 (37.1%) | 140 (40.8%) |  |
| Relationship status |  |  | 0.029^b^ |
| Single | 851 (40.0%) | 229 (45.8%) |  |
| Steady partner | 778 (36.6%) | 154 (30.8%) |  |
| Other status | 497 (23.4%) | 117 (23.4%) |  |
| Gender identity recognition |  |  | <0.001^b^ |
| Yes, always | 326 (14.7%) | 35 (7.3%) |  |
| Sometimes/often | 1337 (60.4%) | 247 (51.4%) |  |
| Never | 551 (24.9%) | 199 (41.4%) |  |
| Living in accordance to gender identity in daily life |  |  | <0.001^b^ |
| Yes | 1325 (59.6%) | 242 (35.1%) |  |
| Partly | 810 (36.4%) | 375 (54.4%) |  |
| No | 90 (4.0%) | 72 (10.4%) |  |
| Fulfilment of medical transition needs |  |  | <0.001^c^ |
| No medical transition desired | 50 (2.4%) | 26 (5.4%) |  |
| Medical transition needs fulfilled | 509 (24.9%) | 60 (12.4%) |  |
| Medical transition needs partially fulfilled | 687 (33.6%) | 116 (23.9%) |  |
| Medical transition needs not fulfilled | 537 (26.3%) | 156 (32.2%) |  |
| Unsure | 259 (12.7%) | 127 (26.2%) |  |
| Living with HIV |  |  | 0.22 ^c^ |
| Yes | 12 (0.6) | 5 (1.2) |  |
| No | 1869 (99.4) | 422 (98.8) |  |
| HIV/STI-protected sex decision-making |  |  | 0.22^b^ |
| Feeling unable | 228 (12.8%) | 37 (10.4%) |  |
| Feeling able | 1556 (87.2%) | 318 (89.6%) |  |

^a^ Percentages per row calculated above the total without missing values for each variable; ^b^ Chi-square test; ^c^ Fisher test
