# Supplementary material for: Discrimination based on gender identity and decision-making regarding HIV/STI-protected sex, a cross-sectional study among trans and non-binary people in Germany
Source: BMC Public Health. 2024 Oct 31;24:3013. doi: 10.1186/s12889-024-20464-2 (PMC11526635; doi:10.1186/s12889-024-20464-2)
Supplement: Supplementary file 4 — Appendix 4. Table A2. Comparison of participant characteristics and discrimination based on gender identity among participants of the TASGstudy with and without missing values for HIV/STI-protected sex decision-making, Germany 2022. [file 12889_2024_20464_MOESM4_ESM.docx]

**Discrimination based on gender identity and decision-making regarding HIV/STI-protected sex, a cross-sectional study among trans and non-binary people in Germany**

**Appendix 4**

**Table A2.** **Comparison of participant characteristics and discrimination based on gender identity among participants of the TASG study with and without missing values for HIV/STI-protected sex decision-making, Germany 2022.**

|  | HIV/STI-protected sex decision-making | |  |
| --- | --- | --- | --- |
|  | No-missing (N=2139) | Missing (N=938) | p-value |
|  | n (%)* | n (%)* |  |
| Gender identity |  |  | <0.001^b^ |
| Female spectrum | 428 (20.0) | 249 (26.5) |  |
| Male spectrum | 481 (22.5) | 191 (20.4) |  |
| Non-binary female spectrum | 266 (12.4) | 117 (12.5) |  |
| Non-binary male spectrum | 301 (14.1) | 89 (9.5) |  |
| Non-binary | 591 (27.6) | 241 (25.7) |  |
| Other | 72 (3.4) | 51 (5.4) |  |
| Age group |  |  | 0.030^c^ |
| 18-29 years | 1,298 (60.7) | 582 (62.0) |  |
| 30-39 years | 555 (25.9) | 216 (23.0) |  |
| 40-49 years | 194 (9.1) | 77 (8.2) |  |
| 50-59 years | 76 (3.6) | 50 (5.3) |  |
| 60 years or older | 16 (0.7) | 13 (1.4) |  |
| Size of place of residence |  |  | 0.003^b^ |
| City with more than 100000 inhabitants | 1,336 (63.9) | 518 (57.2) |  |
| Town/City with less than 100000 inhabitants | 559 (26.7) | 284 (31.4) |  |
| Countryside or village | 197 (9.4) | 103 (11.4) |  |
| Monthly income |  |  | 0.030 ^b^ |
| No income | 94 (5.8) | 28 (9.4) |  |
| ≤2000€ | 1,216 (74.5) | 222 (74.5) |  |
| >2000€ | 322 (19.7) | 48 (16.1) |  |
| Education level |  |  | <0.001^b^ |
| Low | 229 (13.8) | 68 (21.3) |  |
| Medium | 787 (47.3) | 151 (47.2) |  |
| High | 649 (39.0) | 101 (31.6) |  |
| Relationship status |  |  | <0.001^b^ |
| Single | 739 (34.7) | 341 (68.9) |  |
| Steady partner | 836 (39.2) | 96 (19.4) |  |
| Other status | 556 (26.1) | 58 (11.7) |  |
| Gender identity recognition |  |  | 0.250^b^ |
| Yes, always | 272 (13.3) | 89 (13.8) |  |
| Sometimes/often | 1,221 (59.6) | 363 (56.1) |  |
| Never | 555 (27.1) | 195 (30.1) |  |
| Living in accordance to gender identity in daily life |  |  | <0.001^c^ |
| Yes | 1,177 (56.6) | 390 (46.8) |  |
| Partly | 817 (39.3) | 368 (44.2) |  |
| No | 87 (4.2) | 75 (9.0) |  |
| Fulfilment of medical transition needs |  |  | <0.001^c^ |
| No medical transition desired | 50 (2.6) | 26 (4.2) |  |
| Medical transition needs fulfilled | 457 (23.9) | 112 (18.1) |  |
| Medical transition needs partially fulfilled | 621 (32.5) | 182 (29.4) |  |
| Medical transition needs not fulfilled | 507 (26.6) | 186 (30.1) |  |
| Unsure | 274 (14.4) | 112 (18.1) |  |
| Living with HIV |  |  | 0.75^c^ |
| Yes | 14 (0.7) | 3 (0.8) |  |
| No | 1937 (99.3) | 381 (99.2) |  |
| Frequent discrimination based on gender identity |  |  | 0.63^b^ |
| No | 1,394 (78.1) | 388 (77.1) |  |
| Yes | 390 (21.9) | 115 (22.9) |  |

^a^ Percentages per row calculated above the total without missing values for each variable; ^b^ Chi-square test; ^c^ Fisher test
